# Identification of QTLs Controlling α-Glucosidase Inhibitory Activity in Pepper (*Capsicum annuum* L.) Leaf and Fruit Using Genotyping-by-Sequencing Analysis

**DOI:** 10.3390/genes11101116

**Published:** 2020-09-23

**Authors:** Doie Park, Geleta Dugassa Barka, Eun-Young Yang, Myeong-Cheoul Cho, Jae Bok Yoon, Jundae Lee

**Affiliations:** 1 Department of Horticulture, Institute of Agricultural Science & Technology, Jeonbuk National University, Jeonju 54896, Korea; doie9412@naver.com (D.P.); turadugassa@yahoo.com (G.D.B.); 2 Vegetable Research Division, National Institute of Horticultural and Herbal Science, Rural Development Administration, Wanju 55365, Korea; yangyang2@korea.kr (E.-Y.Y.); chomc@korea.kr (M.-C.C.); 3 Research and Development Unit, Pepper and Breeding Institute, K-Seed Valley, Gimje 54324, Korea; yoonjb2@snu.ac.kr

**Keywords:** AGI, *Capsicum annuum*, fruit, GBS, leaf, QTL

## Abstract

Diabetes mellitus, a group of metabolic disorders characterized by hyperglycemia, is one of the most serious and common diseases around the world and is associated with major complications such as diabetic neuropathy, retinopathy, and cardiovascular diseases. A widely used treatment for non-insulin-dependent diabetes is α-glucosidase inhibitors (AGIs) such as acarbose, which hinders hydrolytic cleavage of disaccharides and retard glucose absorption. The ability to inhibit α-glucosidase activity has been reported in leaf and fruit of pepper (*Capsicum annuum* L.). In this study, we aimed to identify quantitative trait loci (QTLs) controlling α-glucosidase inhibitory activity (AGI activity) in pepper leaf and fruit using enzyme assay and genotyping-by-sequencing (GBS) analysis. The AGI activity at three stages of leaf and one stage of fruit development was analyzed by 96 F_2_ individuals. GBS analysis identified 17,427 SNPs that were subjected to pepper genetic linkage map construction. The map, consisting of 763 SNPs, contained 12 linkage groups with a total genetic distance of 2379 cM. QTL analysis revealed seven QTLs (*qAGI1.1*, *qAGI11.1*, *qAGI5.1*, *qAGI9.1*, *qAGI12.1*, *qAGI5.2*, and *qAGI12.2*) controlling AGI activity in pepper leaf and fruit. The QTLs for AGI activity varied by plant age and organ. This QTL information is expected to provide a significant contribution to developing pepper varieties with high AGI activity.

## 1. Introduction

Diabetes mellitus, a group of metabolic disorders characterized by hyperglycemia, is an important human disease around the world [1]. Diabetes results from defective insulin secretion, insulin resistance, or excessive glucagon secretion [2], and eventually causes severe complications by disrupting the metabolism of carbohydrates, proteins, and lipids [3]. Extended exposure to high blood sugar due to diabetes increases the risk of kidney disease, blindness, nerve damage, and blood vessel damage [4], and often contributes to heart disease by doubling the risk of cardiovascular disease [5]. Coronary artery disease leads to the deaths of more than 70% of diabetics older than 65 years [6]. In addition, several microvascular complications of diabetes have been reported, including retinopathy, neocortex, diabetic neurosis, and sexual dysfunction [7].

Diabetes is one of the biggest challenges in the 21st century, as the number of people with diabetes worldwide is expected to rise to 642 million by 2040 [8,9,10]. Diabetes is divided into two types: type 1 diabetes, known as the insulin-dependent type, is an autoimmune disorder that destroys insulin-producing islet cells in the pancreas, and type 2 diabetes, which is the insulin-independent type in which the cells release insulin, but it is nonfunctional due to resistance caused by disruption of the insulin receptor [2,11]. Type 2 diabetes is an acquired disease and currently accounts for 90% of diabetes patients worldwide [12].

To prevent hyperglycemia, it is important to control blood sugar level. One of the therapeutic approaches used to control postprandial hyperglycemia is to retard digestion of sugars by inhibiting digestive enzymes such as α-amylase and α-glucosidase [13,14]. α-glucosidase, located in the brush border of the small intestine, catalyzes the end step of digestion of starch and disaccharides and selectively hydrolyzes terminal (1→4)-linked α-glucose residues to release a single α-glucose molecule, which can be absorbed by intestinal epithelial cells [15]. Molecular compounds that inhibit α-glucosidase, called α-glucosidase inhibitors (AGIs), are used as oral anti-diabetic drugs for type 2 diabetes [16].

Insulin is used to treat type 2 diabetes, but it causes side effects such as insulin resistance or fatty liver [17]. Synthetic oral hypoglycemic agents also cause side effects such as blood problems and liver and kidney strain [18,19]. Three AGIs, acarbose [20], voglibose [14], and miglitol [21], have been developed. However, natural AGIs from plants, which effectively decrease postprandial hyperglycemia, have fewer side effects than insulin, microorganism-originated AGIs, or synthetic agents [22]. Therefore, it is necessary to identify novel α-glucosidase inhibitory compounds derived from natural sources. Many α-glucosidase inhibitory compounds, including terpenes, alkaloids, quinines, flavonoids, phenols, phenylpropanoids, sterides, and other types of compounds, have been reported in medicinal plants [3,23] and vegetable crops [15].

Several pepper (*Capsicum annuum* L.) extracts are reported to have high inhibitory effect on α-glucosidase activity [24]. An investigation on α-glucosidase inhibitory activity (AGI activity) of various pepper extracts from pericarp, placenta, and stalk showed high inhibitory activity in extracts from pericarp and placenta [25]. In addition, pepper leaf extracts showed in vitro inhibitory activity of both α-glucosidase and α-amylase [26]. The AGI activity of pepper fruits depends on genotype [27]. Even though many secondary metabolites are related to AGI activity [15,23], the specific compounds, associated with the activity in pepper, have not been revealed and largely remain obscure.

Four flavonoids, luteolin, amentoflavone, luteolin 7-*O*-glucoside, and daidzein, were found to be strong inhibitors against α-glucosidase in tests of 21 naturally occurring flavonoids [28]. Luteolin 7-*O*-glucoside, isolated from the water extract of pepper leaves, showed α-glucosidase and α-amylase inhibitory activity [26]. However, there was no significant difference in the level of luteolin 7-*O*-glucoside between pepper fruits with high and low activity of AGI [27]. These results suggest that the inhibitory activity of pepper fruits might be caused by compounds other than luteolin 7-*O*-glucoside, and the activity in leaves and fruits might originate from separate compounds. However, studies on molecular compounds and genetic inheritance for AGI activity in pepper leaves and fruits are limited.

Many trait-linked/gene-based markers for diverse disease resistance were developed and are used for marker-assisted selection (MAS) in pepper [29]. MAS can accelerate the development of new varieties [30]. Desired alleles for all genes of agronomic importance can be easily/fastly/accurately introduced into an elite line through a breeding-by-design approach using DNA markers [31]. Quantitative trait loci (QTLs) mapping analysis is highly required because many important agronomic traits such as yield and functional compound content are quantitatively controlled [32].

Therefore, the aim of the present study was to identify QTLs controlling AGI activity in the extracts of pepper leaves and fruits using an F_2_ segregant population and genotyping-by-sequencing (GBS) analysis.

## 2. Materials and Methods

### 2.1. Plant Materials

A segregating F_2_ population consisting of 96 pepper individuals was sown in December 2018 and was grown in a greenhouse of the National Institute of Horticultural and Herbal Science, Rural Development Administration, Wanju-si, Jellabuk-do, Korea, until October 2019. This population was constructed by self-pollination of a Korean-typed pepper (*C. annuum*) F_1_ hybrid obtained from a cross between the cytoplasmic-male sterile maternal line ‘M5’ with low AGI activity and the paternal line ‘AG13-3’ with high AGI activity derived from a pepper cultivar ‘Wonki No. 1’ [33]. A total of 96 F_2_ plants was subjected to DNA extraction for GBS-based genetic linkage mapping. Ten grams of leaves from each plant were sampled; each in April (fourth month after sowing), July (seventh month), and October (tenth month) 2019 to analyze AGI activity. Similarly, 10 g of fruits were collected in October 2019. The leaves and fruits were washed to remove contaminants and dried at 55 °C for 24 h. The dried samples were ground into powder and stored at 4 °C until use.

### 2.2. Analysis of AGI Activity in Pepper Leaves and Fruits

AGI activity was analyzed by an enzyme-substrate interaction in which α-glucosidase from *Saccharomyces cerevisiae* (Sigma-Aldrich, St. Louis, MO, USA) was used as the enzyme and *p*-nitrophenyl glucopyranoside (*p*NPG) (Sigma-Aldrich, St. Louis, MO, USA) was used as the substrate. Acarbose (Sigma-Aldrich, St. Louis, MO, USA) was used as a control for AGI activity. The inhibitory effect of pepper leaf/fruit extracts on α-glucosidase activity was determined using the method described by Kim et al. [27] with modification to exclude the interference of chlorophyll. The improved method was as follows.

First, 250 mg of dried pepper leaves or fruits and 70 mL of 70% ethyl alcohol were mixed and incubated at 55 °C for 16 h. The extract was filtered through a PD-10 Column (GE Healthcare, Chicago, IL, USA), and 1 mL of the filtered extract was concentrated using the speed vacuum centrifuge evaporator CVE-2200 (SUNIL EYELA, Sungnam, Korea). The dried pellet was dissolved in 500 μL of 20 mM sodium phosphate buffer (pH 6.9). The dissolved pellet was filtered using a polyvinylidene fluoride (PVDF) syringe filter (Hyundai Micro, Seoul, Korea). Each sample was extracted in triplicate, and the dried samples were stored at 4 °C until use.

AGI activity was analyzed using 50 μL of pepper leaf or fruit extract, to which 200 μL of α-glucosidase (1 unit·mL^−1^) was added and incubated at 37 °C for 10 min. Next, 400 μL of the substrate (3.0 mM *p*NPG dissolved in 20 mM phosphate buffer) was added to start the reaction. The total reaction volume was 650 μL. The mixture was incubated at 37 °C for 20 min. Finally, 4.35 mL of 0.1 M Na_2_CO_3_ was added to stop the reaction before measuring the absorbance.

Absorbance values at 405 nm wavelength were measured with 200 μL of the total reactant using a microplate spectrophotometer, Epoch (BioTek Instruments, Inc., Winooski, VT, USA). The absorbance value of a control sample, which was prepared by adding 4.95 μL of Na_2_CO_3_ to 50 μL of unreacted extract sample, was measured. The AGI activity was calculated by the following formula.

(1) $$AGI\;activity\;\left(\%\right)=\frac{\left[\left(ABScontrol-ABSblank\right)-\left(ABSsample-ABSsamplecontrol\right)\right]}{\left(ABScontrol-ABSblank\right)}\times 100$$

* *ABScontrol*: Absorbance of the no-inhibitor (α-glucosidase + *p*NPG)
* *ABSblank*: Absorbance of the blank (triple-distilled water)
* *ABSsample*: Absorbance of the sample after reaction (α-glucosidase + *p*NPG + pepper leaf or fruit extract)
* *ABSsamplecontrol*: Absorbance of the sample without reaction (pepper leaf or fruit extract)

### 2.3. Statistical Analysis

Boxplots were drawn using R package ver. 4.0.2 [34]. Correlation analysis between four data sets was performed using R package ver. 3.6.3 based on Pearson’s correlation coefficient. The correlation chart was generated by the package ‘PerformanceAnalytics’ of R program [35].

### 2.4. DNA Extraction

Genomic DNA was extracted from young leaves of F_2_ pepper individuals according to the method described by Lee and Lee [36]. The extracted DNA was dissolved in 100 μL of distilled water with 0.1 μL of 10 mg·mL^−1^ RNase solution (Bio Basic Canada Inc., Markham, ON, Canada). The DNA concentration was measured using BioDrop LITE (BioDrop UK Ltd., Cambridge, UK) and adjusted to 100 ng·μL^−1^. The DNA quality was checked with 1.5% agarose gel electrophoresis and subjected to GBS analysis.

### 2.5. GBS Analysis

DNA samples from the 96 F_2_ individuals were used for the construction of the GBS library according to the method by Eun et al. [37]. The prepared libraries were sequenced with a paired-end read method using HiSeq X (Illumina, San Diego, CA, USA). The raw read data were processed as follows: the sequences of the 96 samples were demultiplexed according to barcode sequences; the sequences of barcode and adapters were removed with the program Cutadapt ver. 1.8.3 [38], and low-quality sequences were trimmed using DynamicTrim and LengthSort programs of SolexaQA ver. 1.13 package [39]. The cleaned data were aligned to the pepper reference genome (*C. annuum* cv. CM334 ver. 1.55) [40], http://peppergenome.snu.ac.kr/, using the Burrows-Wheeler Alignment (BWA) ver. 0.6.1-r104 program [41]. Raw SNPs were detected using the SAMtools ver. 0.1.16 program [42], validated using a SEEDERS *in-house* script [43], and used to generate an integrated SNP matrix.

### 2.6. Genetic Linkage Mapping

Genetic linkage maps were constructed using the JoinMap^®^ ver. 4.1 program (Kyazma B.V., Wageningen, The Netherlands) under the condition of logarithm of the odds (LOD) 3.0 or higher with a maximum distance of 30 cM. Marker grouping was made considering a LOD 3.0 or higher. Map distance was calculated using the Kosambi mapping function [44]. The mapping algorithm used was regression mapping. The population type was F2. The classification type code was a, h, and b. Skewed SNPs were excluded by chi-squared tests (*p* < 0.001), and the markers showing identical segregation or more than ten missing data points were eliminated. Linkage maps were drawn using the MapChart ver. 2.2 program [45].

### 2.7. QTL Analysis

The Windows QTL Cartographer ver. 2.5 program [46] was used for the identification of QTLs. Composite interval mapping (CIM) was performed under 2.0 cM walking speed to evaluate the association between genome-wide markers and traits for AGI activity. The LOD threshold was used at a significance level of 5% by 1000 permutation tests.

## 3. Results

### 3.1. α-Glucosidase Inhibitory Activity in Leaves and Fruits of F_2_ Pepper Plants

**Figure 1:**
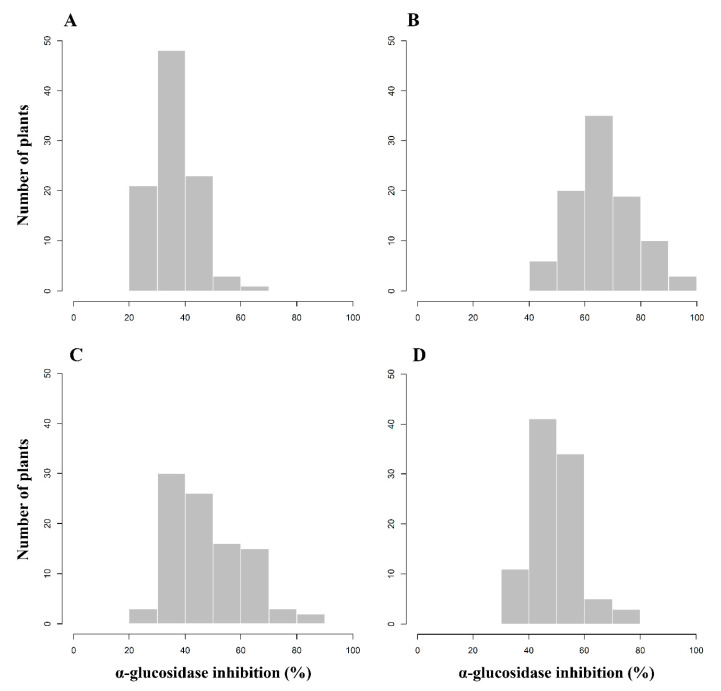
Frequency distribution of 96 F_2_ plants derived from *C. annuum* ‘M5’ × ‘AG13-3’ according to α-glucosidase inhibitory activity analyzed in leaf samples in April (**A**), July (**B**), and October (**C**) and fruit samples in October (**D**).

The α-glucosidase activity assay was conducted using leaf and fruit samples of 96 F_2_ pepper plants. Pepper leaves were collected at three developmental stages (in April, July, and October), while pepper fruits were sampled at only one stage (in October) (Figure 1). Each sample was analyzed in three replicates, and the average value was used for further analysis.

The frequency distribution of pepper plants according to AGI activity is shown in Figure 1. The AGI activity in leaves sampled in April ranged from 25.07% to 65.57%, with an average activity of 36.18% (Figure 1A). The AGI activity in leaf extracts collected in July ranged from 42.56% to 94.09%, with the mean activity of 66.61% (Figure 1B). The inhibitory activity in leaves and fruits analyzed in October ranged from 25.89% to 86.60% and from 30.67% to 77.94%, respectively, with averages of 47.29% and 49.43% (Figure 1C,D). The frequency distributions of the F_2_ pepper population imply that AGI activity is controlled quantitatively not qualitatively.

The distributions of inhibitory activity were compared between four samples: leaves in April, July, and October and fruits in October (Figure S1 (Supplementary Materials)). The activity in leaves in July was the highest, and in April was the lowest. The average activities in leaves and fruits in October were similar, but the activity in leaves had a wider range than that of the activity in fruits.

**Figure 2:**
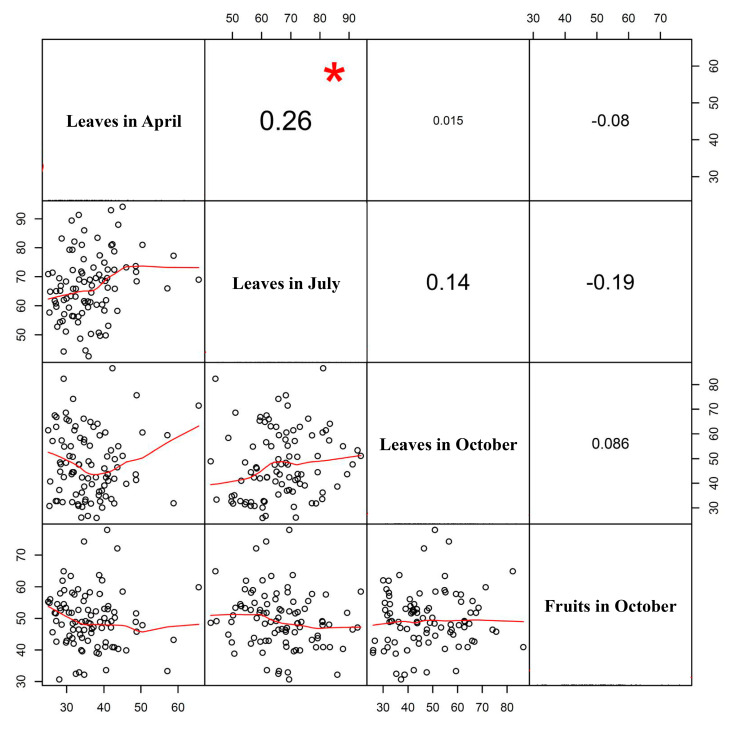
Correlation analysis of α-glucosidase inhibitory activity between four samples, leaves in April, July, and October, and fruits in October, in an ‘M5’ × ‘AG13-3’ F_2_ population of *C. annuum*. Red asterisk indicates significance at *p* < 0.05.

There were no correlations between the four samples for AGI activity except the low correlation between leaves in April and July (Figure 2). The AGI activity in leaves sampled in April was correlated significantly with that of leaves in July (*p* < 0.05) (Figure 2).

### 3.2. SNP Identification and Genotyping Using GBS Analysis

A total of 96 F_2_ pepper plants was subjected to GBS analysis for high-throughput single-nucleotide polymorphism (SNP) detection and genotyping (Table S1 (Supplementary Materials)). A total of 149 Gbp sequences, generated from 987 million raw reads, was obtained by Illumina HiSeq X paired-end read sequencing (Table S1 (Supplementary Materials)). The generated sequence data were classified into 96 samples through a demultiplexing process using a barcode sequence. As a result, 639 million raw reads (64.7%) were demultiplexed (Table S1 (Supplementary Materials)). Subsequently, adapters, barcodes, and noisy sequences were removed by trimming. The total length of trimmed reads was 69 Gbp, which is 46% of the total raw reads. The clean reads were mapped to the reference pepper genome (*C. annuum* cv. CM334 ver. 1.55; http://peppergenome.snu.ac.kr/) [40,47] and subjected to statistical analysis. The total number of mapped reads was 508,353,428, which is 51.5% of the raw reads, and the total number of mapped regions was 11,678,098 (Table S1 (Supplementary Materials)). The average depth of the mapped regions was 13.94, and the total length of mapped regions was 30.7 Mbp, which covers 1.12% of the reference pepper genome (2.8 Gbp). A total of 581,920 SNPs was detected, and 17,427 SNPs were finally selected after filtering based on minor allele frequency > 5% and missing data < 30% (Table S1 (Supplementary Materials)). The final SNP matrix is shown in Table S2.

### 3.3. Genetic Linkage Mapping in an F_2_ Pepper Population

A genetic linkage map of pepper was constructed using the SNP matrix from GBS analysis (Figure S2 (Supplementary Materials)). The genetic map, covering a total genetic distance of 2379.0 cM, consisted of 12 linkage groups, one for each chromosome, and a total of 763 SNPs (Table S3 (Supplementary Materials)). The average distance per linkage group was 198.25 cM, and the average marker interval was 3.1 cM (Table S3 (Supplementary Materials)). The number of markers per linkage group ranged from 50 to 85 SNPs, with an average number of 63.6 markers (Table S3 (Supplementary Materials)). The shortest linkage group was chromosome 8 (109.9 cM), and the longest was chromosome 3 (301.5 cM) (Figure S2 (Supplementary Materials) and Table S3 (Supplementary Materials)). The detailed map information is shown in Table S4 (Supplementary Materials).

### 3.4. Identification of QTLs Controlling AGI Activity in Pepper Leaves and Fruits

**Figure 3:**
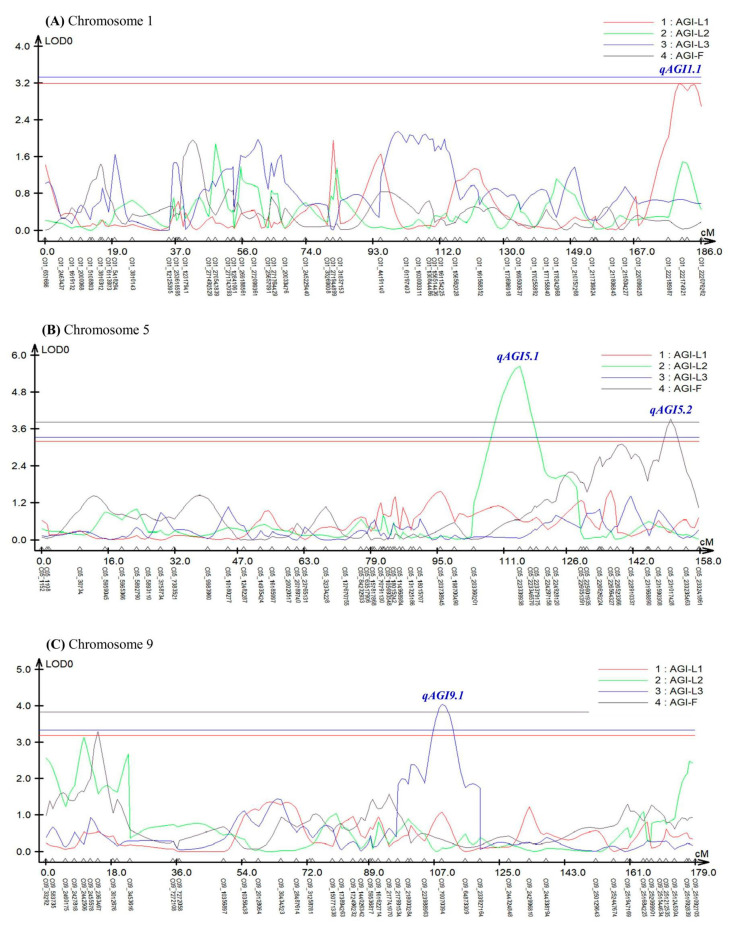

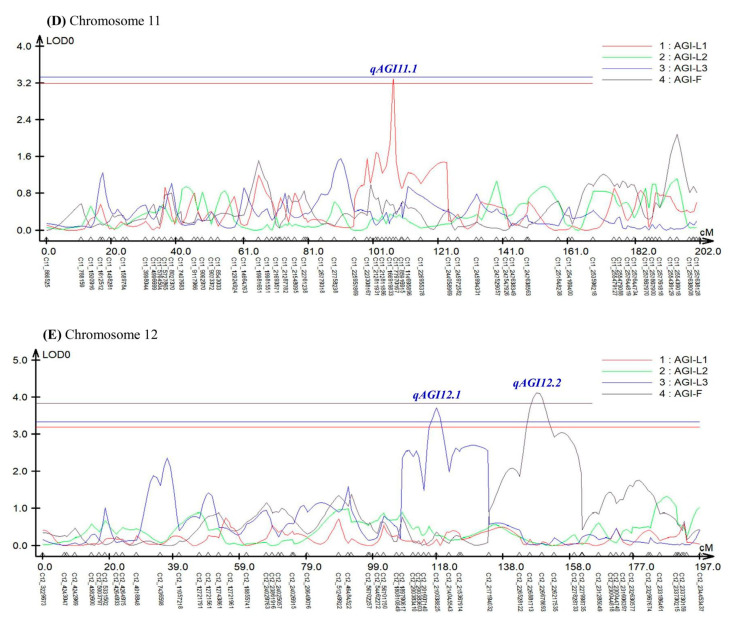
An LOD graph of seven QTLs detected by composite-interval mapping (CIM) analysis for α-glucosidase inhibitory activity in pepper leaves and fruits. AGI-L1, leaves in April; AGI-L2, leaves in July; AGI-L3, leaves in October; AGI-F, fruits in October. (**A**) *qAGI1.1* for AGI-L1 was identified on chromosome 1. (**B**) *qAGI5.1* for AGI-L2 and *qAGI5.2* for AGI-F were positioned on chromosome 5. (**C**) *qAGI9.1* for AGI-L3 was located on chromosome 9. (**D**) *qAGI11.1* for AGI-L1 was found on chromosome 11. (**E**) *qAGI12.1* for AGI-L3 and *qAGI12.2* for AGI-F were mapped on chromosome 12.

Composite interval mapping (CIM) analysis using the software Windows QTL Cartographer ver. 2.5 [46] revealed seven QTLs controlling AGI activity in pepper leaves and fruits (Figure 3 and Table 1).

**Figure 4:**
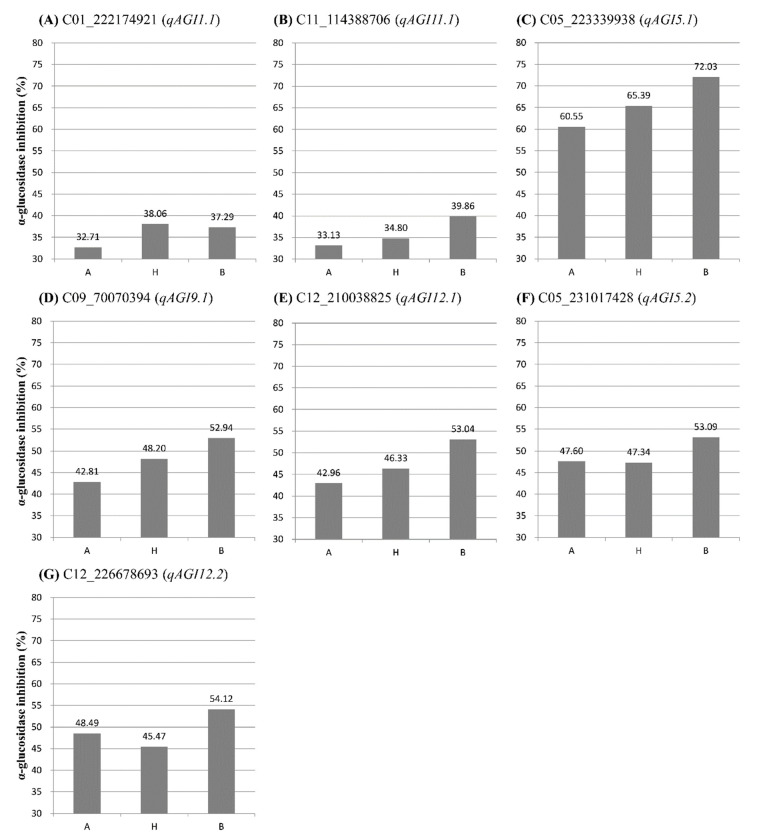
Comparison of average α-glucosidase inhibitory activity between genotypes of the SNPs closely linked to one of the seven QTLs detected in this study. A, maternal genotype; B, paternal genotype; H, heterozygous genotype. (**A**) H and B genotypes of a SNP C01_222174921 linked to the QTL *qAGI1.1* showed higher AGI activity than A genotype. (**B**) B genotypes of C11_114388706 linked to *qAGI11.1* showed higher AGI activity than A and B genotypes. (**C**) A SNP C05_223339938 linked to *qAGI5.1* showed an additive effect on AGI activity depending on genotypes. (**D**) A SNP C09_70070394 linked to *qAGI9.1* showed an additive effect on AGI activity depending on genotypes. (**E**) B genotype of C12_210038825 linked to *qAGI12.1* showed higher AGI activity than A and B genotypes. (**F**) B genotype of C05_231017428 linked to *qAGI5.2* showed higher AGI activity than A and B genotypes. (**G**) B genotype of C12_226678693 linked to *qAGI12.2* showed higher AGI activity than A and B genotypes.

In the experiment using leaves sampled in April, two QTLs (*qAGI1.1* and *qAGI11.1*) were identified at 179.72 cM position on chromosomes 1 and 107.16 cM position on chromosome 11, respectively (Figure 3A,D). The two QTLs, detected with LOD scores of 3.20 and 3.30, showed *R^2^* values of 6.22 and 12.96%, respectively (Table 1). The SNP C01_222174921 was most closely linked to *qAGI1.1*, while C11_114388706 was tightly associated with *qAGI11.1* (Figure 3 and Table 1). The mean differences in AGI activity between maternal (A) and paternal (B) genotypes of two SNPs, C01_222174921 and C11_114388706, were 4.58% and 6.73%, respectively (Figure 4A,B).

A major QTL *qAGI5.1* was detected at 114.70 cM location on chromosome 5 in the leaf samples collected in July, with a LOD score of 5.65 and an explained variance of 15.57% (Table 1). The QTL was positioned between two SNPs: C05_203360201 and C05_223339938. The SNP C05_223339938 was the closest marker (Figure S2 and Table 1), in which the average inhibitory activity in maternal (A) and paternal (B) genotypes was 60.55% and 72.03%, respectively, a difference of 11.48% (Figure 4C).

Two QTLs, *qAGI9.1* and *qAGI12.1*, were detected on chromosomes 9 (109.15 cM) and 12 (118.08 cM), respectively (Figure 3C,E), using the α-glucosidase inhibition data generated from leaves sampled in October (Table 1). The QTL *qAGI9.1*, identified with a LOD score of 4.05 and a coefficient of determination of 8.05, was tightly linked to SNP C09_70070394 (Table 1), in which the difference of average activity was 10.13% (Figure 4D). The QTL *qAGI12.1*, positioned between C12_210038825 and C12_214045543, was detected with a LOD score of 3.72 and an *R*^2^ value of 8.54% (Table 1). The QTL was closely linked to SNP C12_214045543, showing that the average activity (53.04%) in the paternal (B) genotype was higher than that (42.96%) of the maternal (A) genotype (Figure 4E).

Lastly, in the analysis of pepper fruit sampled in October, two QTLs (*qAGI5.2* and *qAGI12.2*) were identified with LOD scores of 3.93 and 4.13 and explained variances of 6.34 and 6.78%, respectively (Table 1). While QTL *qAGI5.2* was located at 150.92 cM on chromosome 5, another QTL (*qAGI12.2*) was positioned at 148.02 cM on chromosome 12 (Figure 3B,E). The QTLs had negative dominance effects of −4.44 and −6.07 (Table 1), meaning that the heterozygous genotypes decreased the AGI activity (Figure 4F,G).

## 4. Discussion

Many people worldwide are suffering and dying due to various complications of diabetes [4,5,6,7]. Even though various diabetes treatments are currently in use, these treatments are associated with a multitude of side effects [17,18,19]. Consequently, it is critical to identify natural AGIs derived from plants for application as diabetes treatments [22]. Many medicinal plants and vegetable crops have been reported to show inhibition of α-glucosidase activity [3,15,23]. Chili pepper is one of the vegetable crops having α-glucosidase inhibitory effects [24]. Previous biochemical analyses showed that pepper leaf and fruit extracts have strong AGI activity [25,26]. However, no reports have examined the inheritance and genetic analysis of AGI activity in pepper. In addition, the identity of secondary metabolites involved in the AGI activity in pepper leaves is not well known, except for luteolin 7-*O*-glucoside [26], and the biosynthetic pathways of AGIs are largely obscure. This is the first study on the inheritance and QTL analysis for AGI activity in pepper.

In this study, we examined the AGI activity in leaves at three plant ages (in April, July, and October) and in fruits in October for 96 F_2_ pepper plants (Figure 1 and Figure S1 (Supplementary Materials)). We compared the AGI activity between organs (leaf and fruit) and plant ages (Figure S1 (Supplementary Materials)). The AGI activity in leaf extracts varied according to plant age, with the highest activity in July (Figure S1 (Supplementary Materials)). This result might be caused by the synthesis of various secondary metabolites having AGI activity. Many AGIs such as flavonoids, alkaloids, terpenoids, anthocyanins, glycosides, phenols, sterides, coumarins, saponins, quinines, phenylpropanoids, and iminosugars, have been reported in plants [3,15,22,23]. Although luteolin 7-*O*-glucoside isolated from pepper leaf extracts was reported to show AGI activity [26], its content was not significant between cultivars with high and low AGI activity in pepper fruits [27]. This result indicated the existence of other unknown compounds that inhibit α-glucosidase activity in pepper fruits.

In the experiment with samples collected in October, the average AGI activity in leaves was similar to that in fruits, but the activity range in leaves was wider than that in fruits (Figure 1 and Figure S1 (Supplementary Materials)). There was no correlation between AGI activities of leaves and fruits sampled in October (Figure 2). This result implies the possible involvement of different gene products for the biosynthesis of secondary metabolites with AGI activity in leaves and fruits. A corroborating result was obtained from the identification of different QTLs controlling AGI activity in leaves and fruits collected in October (Table 1). These results suggest that AGI activity is controlled by different genes at different stages (in April, July, and October) and in different organs (leaf and fruit). Since the AGI activity inheritance patterns in four analyses varied, we aimed to identify all QTLs controlling AGI activity using four sets of data.

GBS analysis [48] was performed to detect SNPs and analyze the genotypes of the detected SNPs in 96 F_2_ individuals. Approximately, 149 Gbp of DNA sequences were obtained using the Illumina HiSeq X platform (Table S1 (Supplementary Materials)). The amount was large enough for GBS analysis compared with the study of Eun et al. [47]. A total of 17,427 SNPs was detected (Table S1 (Supplementary Materials)) and 763 SNPs were mapped (Table S3). Less than 5% of the SNPs were incorporated into the genetic map due to elimination of SNPs such as identicals, more than ten missing data, and with not significant chi-square values (*p* < 0.001) (Table S4). In addition, ‘Map 1’, which contains a low number of markers, was used for the construction of a more accurate map using the JoinMap program. The constructed pepper genetic map, consisting of 12 linkage groups, covered a total linkage distance of 2379 cM (Figure S2 and Table S3 (Supplementary Materials)). The numbers in SNP names indicate positions (bp) in pepper physical map (Figure S2 (Supplementary Materials)). Overall synteny of marker position was consistent with the previous genetic map of *C. annuum* [40,47]. These results indicate that the SNPs are distributed genome-wide, and the map can be used for QTL analysis according to Eun et al. [37].

QTL analysis revealed seven significant QTLs controlling AGI activity in pepper leaves and fruits (Figure 3 and Table 1). As expected, the identified QTLs were separately located for the four data sets (Figure 3 and Table 1); that is, there was no common QTL. These results might be due to the presence of various secondary metabolites, such as flavonoids, carotenoids, or phenolic compounds, having AGI activity [3,15,23], and to the environmental effect on production and accumulation of the secondary metabolites [49]. Therefore, the detected QTLs might be responsible for the enzymes involved in the biosynthetic pathways of the secondary metabolites. In pepper leaves, luteolin 7-*O*-glucoside, a flavone derivative, was reported to show only α-glucosidase and α-amylase inhibitory activity [26]. Thus, further studies are needed to compare these QTLs with luteolin 7-*O*-glucoside synthesis-related genes, including *CHS* (encoding chalcone synthase), *CHI* (chalcone isomerase), *FNSI* (flavone synthase I), *FNSII* (flavone synthase II), *F3’H* (flavonoid 3’-hydroxylase), and *F7’G* (flavone 7-*O*-β-glucosyltransferase) [50,51].

Wahyuni et al. [51] identified several metabolite QTLs (mQTLs) and gene expression QTLs (eQTLs) related to flavonoid synthesis in pepper. The QTL *qAGI1.1* was located in a similar position to one mQTL (naringenin chalcone), four eQTLs (*FLS*, *CHI-2*, *CHS-2*, and *CHS-1*), and a transcription factor (*Ca-MYB12*). The QTL *qAGI9.1* was positioned on chromosome 9, on which three mQTLs (naringenin chalcone, luteolin-methyl-acetyl-apiofuranosyl-hexose, and luteolin-methyl-*O*-di-hexose) and five eQTLs (*FS-2*, *CHI-2*, *CHI-1*, *CHS-2*, *CHS-1*) also were located [51]. These results imply that the AGI activity in pepper leaves is related to flavonoid synthesis.

Seven SNPs closest to each QTL controlling AGI activity were used to evaluate the selection efficiency of the SNPs (Figure 4 and Table 1). For all the SNPs, an average AGI activity in the B genotype from the paternal parent ‘AG13-3’ was higher than that of the A genotype from the maternal parent ‘M5’ (Figure 4). These results suggest that the SNPs can be used for MAS of pepper lines with high AGI activity. Pepper lines with high AGI activity can be developed in segregating populations by selecting B genotypes for the seven SNPs. Therefore, further study on the development of molecular markers derived from the identified SNPs is required for MAS.

## 5. Conclusions

In this study, we identified seven significant QTLs controlling the AGI activity in pepper leaves and fruits using GBS analysis. In addition, seven SNPs closely linked to one of these QTLs were selected and evaluated for MAS. The information of the QTLs and QTL-linked SNPs is expected to accelerate pepper breeding for the development of varieties with high AGI activity. Furthermore, this study provides highly valuable data to identify secondary metabolites with AGI activity in pepper.

## Figures and Tables

**Table 1:** Detailed information of QTLs controlling α-glucosidase inhibitory activity in pepper leaves and fruits.

Sample Type and Collection Time	QTL	Marker Interval of QTL Region	QTL Peak Position (cM)	Additive Effect	Dominance Effect	*R*^2^^a^ (%)	LOD ^b^ Score	LOD ^c^ Threshold
Leaves in April	*qAGI1.1*	C01_222185987-C01_222174921	179.72	−3.67	3.34	6.22	3.20	3.18
	*qAGI11.1*	C11_114388706-C11_77970167	107.16	−2.97	−2.27	12.96	3.30	3.18
Leaves in July	*qAGI5.1*	C05_203360201-C05_223379175	114.70	−4.87	−4.06	15.57	5.65	3.34
Leaves in October	*qAGI9.1*	C09_70070394-C09_64873309	109.15	−6.72	0.63	8.05	4.05	3.34
	*qAGI12.1*	C12_203983726-C12_214045543	118.08	−5.84	−0.34	8.54	3.72	3.34
Fruits in October	*qAGI5.2*	C05_231017428-C05_233235463	150.92	−4.75	−4.44	6.34	3.93	3.85
	*qAGI12.2*	C12_226881713-C12_226678693	148.02	−1.25	−6.07	6.78	4.13	3.85

^a^*R*^2^, proportion of variance explained by QTL at the test site. ^b^ LOD, logarithm of the odds. ^c^ LOD threshold was determined by 1000-permutation tests.

## Supplementary Materials

The following are available online at https://www.mdpi.com/2073-4425/11/10/1116/s1, Figure S1: Boxplots for α-glucosidase inhibitory activity in leaf extracts in April, July, and October and fruit extracts in October in an ‘M5’ × ‘AG13-3’ F_2_ population of *Capsicum annuum*. Figure S2: Genetic linkage maps constructed in an F_2_ population of *Capsicum annuum* ‘M5’ (low activity) × ‘AG13-3’ (high activity) through genotyping-by-sequencing analysis. Table S1: Summary of sequence data generated by genotyping-by-sequencing analysis. Table S2: The SNP matrix generated by GBS analysis. Table S3: Summary of the pepper genetic linkage map constructed for an ‘M5’ × ‘AG13-3’ F_2_ population of *Capsicum annuum*. Table S4: Detailed map information consisting of 763 SNPs in an F_2_ population from *Capsicum annuum* ‘M5’ × ‘AG13-3.’

## Abbreviations

AGI activity	α-glucosidase inhibitory activity
AGI	α-glucosidase inhibitor
CIM	composite interval mapping
eQTL	gene expression QTL
GBS	genotyping-by-sequencing
LOD	logarithm of the odds
MAS	marker-assisted selection
mQTL	metabolite QTL
*p*NPG	*p*-nitrophenyl glucopyranoside
QTL	quantitative trait loci
SNP	single-nucleotide polymorphism
