# Supplementary material for: Identification of QTLs Controlling α-Glucosidase Inhibitory Activity in Pepper (Capsicum annuum L.) Leaf and Fruit Using Genotyping-by-Sequencing Analysis
Source: Genes (Basel). 2020 Sep 23;11(10):1116. doi: 10.3390/genes11101116 (PMC7650571; doi:10.3390/genes11101116)
Supplement: Supplementary file 1 [file genes-11-01116-s001.zip › Figure S1. Boxplots for α-glucosidase inhibitory activity in leaf extracts in April, July, and October and fruit extracts.docx]

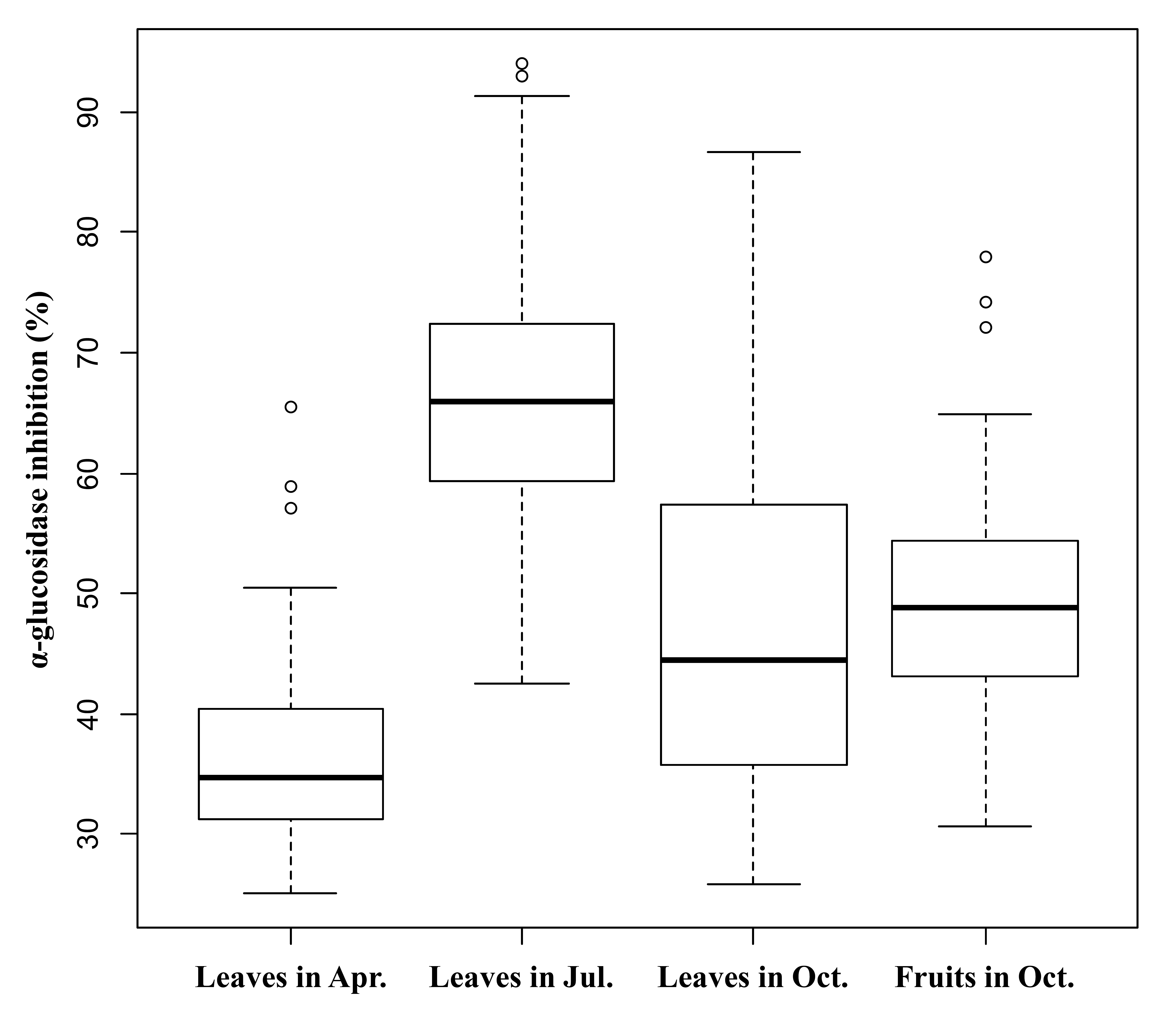


**Figure S1.** Boxplots for α-glucosidase inhibitory activity in leaf extracts in April, July, and October and fruit extracts in October in an ‘M5’ × ‘AG13-3’ F_2_ population of *Capsicum annuum.*
