# Supplementary material for: Identification of QTLs Controlling α-Glucosidase Inhibitory Activity in Pepper (Capsicum annuum L.) Leaf and Fruit Using Genotyping-by-Sequencing Analysis
Source: Genes (Basel). 2020 Sep 23;11(10):1116. doi: 10.3390/genes11101116 (PMC7650571; doi:10.3390/genes11101116)
Supplement: Supplementary file 1 [file genes-11-01116-s001.zip › Figure S2. Genetic linkage maps constructed in an F2 population of Capsicum annuum ‘M5’ (low activity) × ‘AG13-3’ (high activity).docx]

**Figure S2**. *Cont*.

**Figure S2**. Genetic linkage maps constructed in an F_2_ population of *Capsicum annuum* ‘M5’ (low activity) × ‘AG13-3’ (high activity) through genotyping-by-sequencing analysis. Numbers on left, genetic position (centi Morgan, cM); numbers on right, GBS-based SNPs.
