# Supplementary material for: Identification of QTLs Controlling α-Glucosidase Inhibitory Activity in Pepper (Capsicum annuum L.) Leaf and Fruit Using Genotyping-by-Sequencing Analysis
Source: Genes (Basel). 2020 Sep 23;11(10):1116. doi: 10.3390/genes11101116 (PMC7650571; doi:10.3390/genes11101116)
Supplement: Supplementary file 1 [file genes-11-01116-s001.zip › Table S1. Summary of sequence data generated by genotyping-by-sequencing analysis.docx]

**Table S1.** Summary of sequence data generated by genotyping-by-sequencing analysis.

| Summary | Data |
| --- | --- |
| Number of F_2_ plants for multiplexing | 96 |
| Total number of raw reads generated | 986,717,428 (100%) |
| Total base number of raw reads | 148,994,331,628 (149.0 Gbp) |
| Total number of demultiplexed reads | 638,694,784 (64.7%) |
| Total number of trimmed reads | 584,863,916 (59.3%) |
| Total number of mapped reads | 508,353,428 (51.5%) |
| Total number of mapped regions | 11,678,098 |
| Average depth of mapped region | 13.94 |
| Total length of mapped regions | 30,703,724 (30.7 Mbp) |
| Total length of reference pepper genome | 2,753,501,687 (2.8 Gbp) |
| Coverage of reference pepper genome | 1.12% |
| Total number of SNPs detected | 581,920 |
| Total number of SNPs filtered | 17,427 |
