# Supplementary material for: Identification of QTLs Controlling α-Glucosidase Inhibitory Activity in Pepper (Capsicum annuum L.) Leaf and Fruit Using Genotyping-by-Sequencing Analysis
Source: Genes (Basel). 2020 Sep 23;11(10):1116. doi: 10.3390/genes11101116 (PMC7650571; doi:10.3390/genes11101116)
Supplement: Supplementary file 1 [file genes-11-01116-s001.zip › Table S3. Summary of the pepper genetic linkage map constructed for an ‘M5’ × ‘AG13-3’ F2 population of Capsicum annuum.docx]

**Table S3**. Summary of the pepper genetic linkage map constructed for an ‘M5’ × ‘AG13-3’ F_2_ population of *Capsicum annuum*.

| Chromosome | Number of markers | Linkage distance (cM) | Marker density (cM/marker) |
| --- | --- | --- | --- |
| 1 | 59 | 186.1 | 3.2 |
| 2 | 62 | 210.6 | 3.4 |
| 3 | 85 | 301.5 | 3.5 |
| 4 | 50 | 175.7 | 3.5 |
| 5 | 62 | 157.9 | 2.5 |
| 6 | 58 | 252.5 | 4.4 |
| 7 | 78 | 242.1 | 3.1 |
| 8 | 50 | 109.9 | 2.2 |
| 9 | 52 | 178.9 | 3.4 |
| 10 | 60 | 164.9 | 2.7 |
| 11 | 82 | 201.8 | 2.5 |
| 12 | 65 | 197.1 | 3.0 |
| Total | 763 | 2379.0 | 3.1 |
